# Comparison of GFR estimation in patients with diabetes mellitus using the EKFC and CKD-EPI equations

**DOI:** 10.1007/s40620-024-02202-4

**Published:** 2025-01-10

**Authors:** Felix Eisinger, Mareike Neumann, Matthias Wörn, Andreas Fritsche, Nils Heyne, Andreas Peter, Andreas L. Birkenfeld, Reiner Jumpertz von Schwartzenberg, Ferruh Artunc

**Affiliations:** 1 Department of Diabetology, Endocrinology, Nephrology, University of Tuebingen, Tuebingen, Germany; 2 Institute for Diabetes Research and Metabolic Diseases of the Helmholtz Center Munich at the University of Tuebingen, Tuebingen, Germany; 3 German Center for Diabetes Research (DZD E.V.), Neuherberg, Germany; 4 Department for Diagnostic Laboratory Medicine, Institute for Clinical Chemistry and Pathobiochemistry, University Hospital of Tuebingen, Tuebingen, Germany; 5 Southwest Clinic Network, Hospital Sindelfingen, Section Nephrology, Sindelfingen, Germany

**Keywords:** Diabetes mellitus, Creatinine, Cystatin C, Estimated glomerular filtration rate (eGFR), Chronic Kidney Disease-Epidemiology Collaboration (CKD-EPI), European Kidney Function Consortium (EKFC)

## Abstract

**Background:**

The estimation of glomerular filtration rate (eGFR) is essential in the early detection of diabetic nephropathy. We herein compare the performance of common eGFR formulas against a gold standard measurement of GFR in patients with diabetes mellitus.

**Methods:**

GFR was measured in 93 patients with diabetes mellitus using iohexol clearance as the reference standard. The performance of the creatinine- and cystatin C-based EKFC formulas (2021, 2023) and the CKD-EPI formulas (2009, 2012) was compared against measured GFR.

**Results:**

Sixty patients with type 2 diabetes mellitus and 33 patients with type 1 diabetes mellitus were included. The creatinine-based EKFC formula showed lower bias and higher accuracy than the CKD-EPI formula. No significant difference was observed between the cystatin C-based formulas. The combined creatinine- and cystatin C-based formulas had the highest accuracy and lowest bias. Body fat or diabetes type did not significantly influence the accuracy of the cystatin C-based formulas.

**Conclusions:**

Our study demonstrated a slight advantage of the creatinine-based EKFC formula over the CKD-EPI formula in patients with diabetes. However, both for the CKD-EPI and the EKFC formula, the best performance was achieved by the combined creatinine- and cystatin C-based formulas.

**Graphical abstract:**

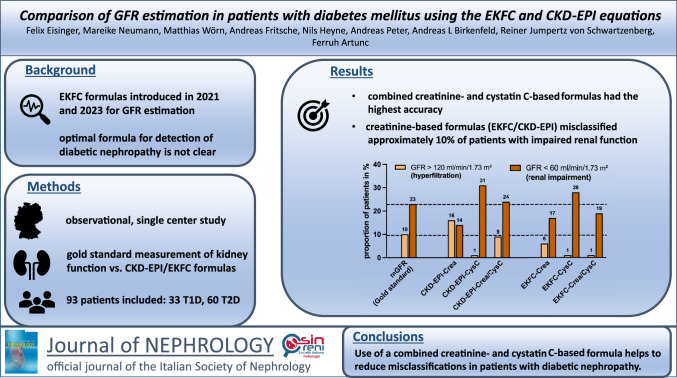

**Supplementary Information:**

The online version contains supplementary material available at 10.1007/s40620-024-02202-4.

## Introduction

Despite great advances in the treatment of diabetes mellitus, diabetic nephropathy is still the leading cause of end-stage kidney disease (ESKD) in Western countries [1]. In a long-term follow up of patients diagnosed with type 1 diabetes mellitus between 1965 and 1980, the incidence of ESKD after 40 years of diabetes was as high as 26.5% [2]. Thus, it remains essential to identify at-risk patients in an early stage of diabetic nephropathy and correctly assess kidney function in these patients. In clinical practice, creatinine and cystatin C are the most commonly used markers to estimate glomerular filtration rate (eGFR). In 2009, the Chronic Kidney Disease Epidemiology Collaboration (CKD-EPI) developed a creatinine-based formula (CKD-EPI_Crea_), which was recommended in the 2012 KDIGO guidelines for the evaluation of chronic kidney disease [3]. In 2012, a CKD-EPI formula based on cystatin C (CKD-EPI_CysC_), respectively, a combination of creatinine and cystatin C (CKD-EPI_Crea-CysC_) was published and has remained an alternative for the estimation of GFR since [4]. While both the CKD-EPI_Crea_ and the CKD-EPI_Crea-CysC_ formula include a race factor, an attempt to provide a novel race-free CKD-EPI formula was made in 2021 [5]. However, the European Federation of Clinical Chemistry and Laboratory Medicine recently recommended not to use this formula in Europe due to a poorer performance than the 2009 formula in most European patients [6]. Instead, the CKD-EPI 2009 formula should be used without applying the race factor. In 2021 and 2023, the European Kidney Function Consortium (EKFC) presented three new, promising equations for the European population based on creatinine (EKFC_Crea_), cystatin C (EKFC_CysC_) and a combination of both (EKFC_Crea-CysC_: mean of the creatinine-based and the cystatin C-based EKFC formula) [7, 8]. Although patients with diabetic nephropathy have been included in the database used for the development of the EKFC formula, no study has yet compared the performance of the three EKFC formulas with the 2009 and 2012 CKD EPI formulas in this subgroup for central European patients against a gold standard method with measurement of GFR using the plasma iohexol clearance. In the present study, we measured GFR using this method in 93 individuals with diabetes mellitus and compared it to both the EKFC and the CKD-EPI formulas. Furthermore, we evaluated potential covariates such as biometric factors, medication, HbA1c and body composition.

## Materials and methods

### Study design

Patients with diabetes mellitus type 1 or 2 were recruited from the diabetes center of the University hospital of Tuebingen between April 2022 and July 2023. Patients with acute kidney injury, acute infections, recent start of a sodium-glucose cotransporter type 2 (SGLT2) inhibitor or a renin–angiotensin–aldosterone system (RAAS) inhibitor (within the last 7 days), known allergy to contrast agents or a latent or manifest hyperthyroidism were excluded from the study. All patients provided written informed consent. The study was approved by the local institutional review board (020/2022BO2) and registered in the German Clinical Trials Register (DRKS00028843).

### Study protocol

The glomerular filtration rate was determined by the gold standard method of plasma iohexol clearance on a single day. The clearance measurement was performed during the day in a range from 10 am to 4 pm. After obtaining a baseline blood sample prior to the injection, the patient received a bolus of 5 or 10 ml of iohexol (Omnipaque 300 mg/ml or Accupaque 240 mg/ml), depending on the availability of iohexol. Afterwards, the peripheral line was flushed with saline. Blood samples (EDTA) for the determination of iohexol clearance rate were taken after 120, 150, 180 and 210 min. Analysis of the blood samples was conducted via high performance liquid chromatography (HPLC) as described below. Iohexol concentration in each blood sample was measured in duplicate. Glomerular filtration rate was calculated using the slope of the logarithmic iohexol concentrations and its intercept. Overestimation was corrected by the formula of Bröchner-Mortensen [9]. The obtained GFR values were normalized for body surface area. Impaired kidney function was defined according to the KDOQI guidelines as GFR < 60 ml/min/1.73 m^2^ [10]. Hyperfiltration was defined as GFR > 120 ml/min/1.73 m^2^ [11, 12] or > 134 ml/min/1.73 m^2^ [13]. During the study visit, medical data of each patient including medical history, medication, height, weight and waist circumference was obtained.

### Determination of the plasma iohexol concentration by HPLC

A detailed description of the iohexol measurement can be found in Supplementary Information.

### Laboratory analyses

The baseline laboratory values were analyzed in the diagnostic laboratory at the Institute for Clinical Chemistry and Pathobiochemistry of the University Hospital of Tuebingen under DAkkS accreditation according to ISO15189. Specifically, plasma and urine creatinine (enzymatic method with calibration according to IDMS) and plasma cystatin C (turbidimetric method) were determined on the ADVIA XPT clinical chemistry analyzer, and urine albumin was determined by nephelometry on the BN ProSpec System (all from Siemens Healthineers, Eschborn, Germany). For the creatinine measurement, an enzymatic ECRE_2 assay was used. In this assay, creatinine is converted to creatine by the action of creatininase. The creatine formed is hydrolyzed by creatinase to produce sarcosine, which is decomposed by sarcosine oxidase to form glycine, formaldehyde, and hydrogen peroxide. In the presence of peroxidase, the hydrogen peroxide formed yields a blue pigment. The creatinine concentration is obtained by measuring the absorbance of the blue color at 596/694 nm. The absorbance of the color is proportional to the creatinine concentration. The plasma cystatin C concentrations were measured using a standardized assay (ADVIA Chemistry CYSC_2 assay standardized to the IFCC reference material ERM-DA471). With regard to the eGFR, the following equations were used: the 2009 CKD-EPI_Crea_ equation without application of the race correction factor, the 2012 version of the CKD-EPI_Crea-CysC_ equation, the 2021 EKFC_Crea_ equation and the 2023 EKFC_CysC_ and EKFC_Crea-CysC_ equation (formulas can be found in Supplementary Information).

### Body composition and volume status assessment

Body composition and volume status were analyzed during the study visit using the Body Composition Monitor (BCM, Fresenius Medical Care) which determines fluid status (overhydration, total body water, extracellular and intracellular water) and body composition (lean and adipose tissue mass). The BCM device was validated against standard reference techniques of body composition measurement [14].

### Statistical analysis

Statistical analysis was performed with the JMP 16.2.0 statistical software package and the IBM-SPSS Version 28.0. Data are given as median with interquartile range or as absolute figures with percentage. Spearman’s correlation analysis was used to test for correlation between measured GFR (mGFR) and eGFR. Bias was defined as the absolute difference between eGFR and mGFR. Accuracy was defined as the percentage of eGFR values within 10% (P10) or 30% (P30) of the mGFR. Precision was defined as the interquartile range (IQR) of the difference between mGFR and eGFR. The Wilcoxon signed-rank test was used to compare eGFR, bias and the influence of metrically-scaled covariates. The McNemar test was applied to compare accuracies. The influence of nominally-scaled covariates on the accuracy of GFR measurement was analyzed with a chi-square test. Univariate pairwise correlation was used to test for the relationship between body composition parameters with GFR results. A *p*-value of less than 5% was set as level of significance.

## Results

### Patient characteristics

Between April 2022 and July 2023, 93 patients with diabetes mellitus were enrolled in this study. Sixty patients had type 2 and 33 had type 1 diabetes. Median age was 57 years [IQR 44.5–65.5] and median HbA1c was 7.9% [7–10.2]. The vast majority (98.9%) of the participants were of Caucasian ethnicity. Further patient characteristics are depicted in Table 1. Results of the body composition monitoring measurement can be found in the Supplemental Table 1. Median body mass index (BMI) was 27.3 [24.8–33.3] kg/m^2^, adipose mass was 46.2 [36.4–60] kg, there was no overhydration.

**Table 1:** Patient characteristics (*n* = 93)

Age (years)	57 [44.5–65.5]
Sex	
Men	61 (65.6%)
Women	32 (34.4%)
Diabetes mellitus	
Type 1	33 (35.5%)
Type 2	60 (64.5%)
Time since initial diagnosis (years)	11 [1.25–18.8]
Height (m)	1.74 [1.65–1.81]
Weight (kg)	87 [73.6–100]
BMI (kg/m^2^)	27.3 [24.8–33.3]
Waist circumference (cm)	104 [90–115]
Comorbidities	
Hypertension	53 (57%)
Coronary artery disease	15 (16.1%)
Medication	
Metformin	49 (52.7%)
SGLT2 inhibitor	42 (45.2%)
DPP-4 inhibitor	8 (8.6%)
GLP1 analogue	35 (37.6%)
Insulin	68 (73.1%)
RAAS inhibitor	59 (63.4%)
Hemoglobin (g/dl)	14.4 [13.3–15.4]
Total cholesterol (plasma) (mg/dl)	162 [133.5–194.8]
CRP (mg/dl)	0.2 [0.04–0.7]
TSH (mU/l)	1.7 [1–2.2]
Blood urea (plasma) (mg/dl)	31 [23–39.5]
HbA1c (mmol/mol)	62.8 [53–88]
HbA1c (%)	7.9 [7–10.2]
Creatinine (plasma) (mg/dl)	0.8 [0.6–1]
Cystatin C (plasma) (mg/l)	1 [0.85–1.3]
Urine albumin-creatinine ratio (mg/g creatinine)	32.3 [15–108.6]

*Data are given as median [interquartile range] or number (percent)*

### Comparison of creatinine-, cystatin- and creatinine-cystatin C-based GFR formulas

**Fig. 1:**
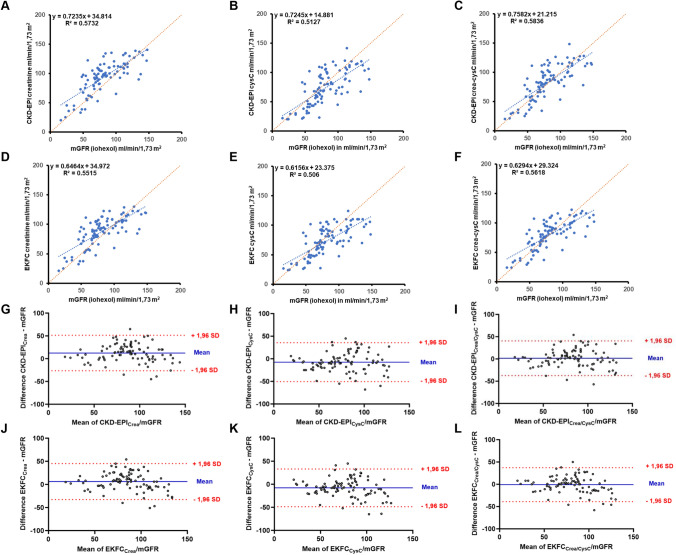
Scatter plot and Bland–Altman-Plot. **A**–**F** Scatter plot depicting the relationship between measured glomerular filtration rate (mGFR) and GFR formulas. The trendline is given in blue, the line of identity is given in orange. Coefficient of determination (*R*^2^) and equation of the trend line are given. **G**–**L** Bland–Altman-Plot illustrating the difference between estimated GFR (eGFR) and mGFR on the *Y* axis and the mean of both measurement on the *X* axis. The mean difference is represented in blue, one standard deviation (SD) is displayed in red

**Fig. 2:**
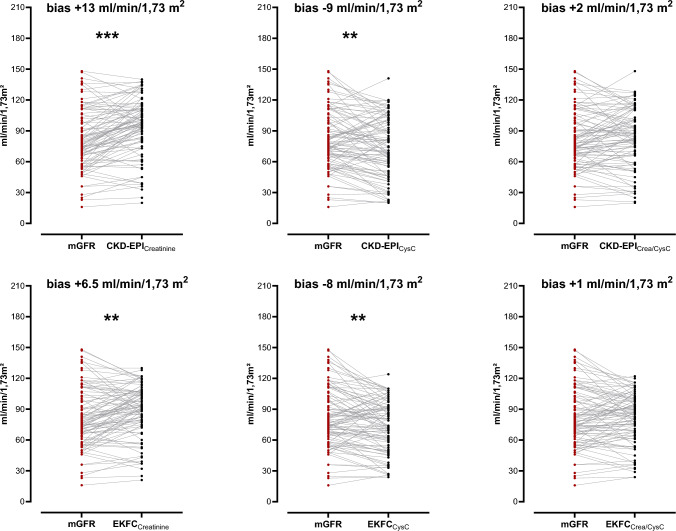
Pairwise comparison of mGFR and eGFR. Pairwise illustration of measured glomerular filtration rate (mGFR) (red) and estimated GFR (eGFR) (blue). Bias was defined as the mean absolute difference between eGFR and mGFR. A Wilcoxon signed rank test for paired samples was used to test for differences between eGFR and mGFR. **P-value ≤ 0.01, ***P-value ≤ 0.001

Median mGFR was 76 [62–102.5) ml/min/1.73 m^2^ in the overall cohort. Both the CKD-EPI and the EKFC formulas showed a high correlation with the measured GFR (correlation coefficients between 0.71 (95% CI: 0.60–0.80) and 0.77 (95% CI: 0.66–0.84), *p* < 0.001, Fig. 1). While both the CKD-EPI_Crea_ (eGFR 98 [80.5–111] ml/min/1.73 m^2^) and EKFC_Crea_ formula (eGFR 92 [76–106] ml/min/1.73 m^2^) showed significantly higher GFR values than the measured GFR, the CKD-EPI_CysC_ (eGFR 74 [50.5–100.5] ml/min/1.73 m^2^) and EKFC_CysC_ formula (eGFR 73 [54–92.5] ml/min/1.73 m^2^) led to significantly lower GFR values compared to the mGFR (*p* < 0.05) (Table 2). The eGFR calculated with the combined creatinine and cystatin C formulas did not differ significantly from the mGFR. Scatter plots and Bland–Altman plots of eGFR values in relation to mGFR values can be found in Fig. 1. While the EKFC_Crea_ and the EKFC_Crea-CysC_ formula led to significantly lower absolute bias compared to the CKD-EPI_Crea_ and the CKD-EPI_Crea-CysC_ formula, no significant difference was found for the cystatin C-based formulas. The EKFC_Crea-CysC_ formula had the lowest bias of all formulas (1 (95% CI: − 4.9–3.1) ml/min/1.73 m^2^) (Fig. 2). For all formulas, bias tended to be lower with increasing age (Supplemental Fig. 2, 3 and 4). The combined creatinine- and cystatin C-based formulas showed the highest P30 accuracy (EKFC P30: 81.7 (95% CI: 72.7–88.3) %; CKD-EPI P30: 82.8 (95% CI: 73.9–89.1) %, Table 2). The cystatin C-based formula differed by more than 30% from the creatinine-based formula in 30 patients (32%) for the CKD-EPI formula and in 16 patients (17%) for the EKFC formula. In these cases, the combined creatinine- and cystatin C-based formula had a P30 accuracy of 94 (95% CI: 71.7–98.9) % (EKFC formula) and 73 (95% CI: 55.6–85.8) % (CKD-EPI formula). The mean difference between the CKD-EPI formula and the EKFC formula was 6.2 (95% CI: 5.2–7.0) ml/min/1.73 m^2^ for the creatinine-based formula, 2.4 (95% CI: 1.1–3.7) ml/min/1.73 m^2^ for the cystatin C-based formula and 0.4 (95% CI: − 0.8–1.5) ml/min/1.73 m^2^ for the combined formula. For the creatinine-based and the combined formula, the difference between the CKD-EPI and the EKFC formula was larger for creatinine values < 0.7 mg/dl and age < 40 years.”

**Table 2:** Bias, accuracy, precision and classification of kidney function

	mGFR	CKD- EPI Crea 2009	CKD- EPI CysC 2012	CKD- EPI Crea-Cys 2012	EKFC Crea 2021	EKFC CysC 2023	EKFC Crea-Cys 2023
GFR (ml/min/1.73 m^2^)	**76 [62–103]**	98 [81–111]	74 [51–101]	85 [66–107]	92 [76–106]	73 [54–93]	85 [66–99]
Bias (ml/min/1.73 m^2^) (95% CI)		13 (8.2–16.3)	-9 (-12–-2.9)	2 (-2.5–5.7)	6.5 (2.1–10.1)	-8 (-3.6–-12.3)	1 (-4.9–3.1)
P30 accuracy (%) (95% CI)		74.2 (64.5–82.0)	67.7 (57.7–76.4)	82.8 (73.9–89.1)	78.5 (70.3–86.5)	69.9 (59.9–78.3)	81.7 (72.7–88.3)
P10 accuracy (%) (95% CI)		31.2 (22.7–41.2)	22.6 (16.2–33.2)	36.6 (27.5–46.7)	34.4 (25.6–44.5)	23.7 (17.1–34.4)	38.7 (29.5–48.9)
Precision (ml/min/1.73 m^2^)		26	24.5	24.5	24	24	21.5
Patients with renal impairment	**21 (22.6%)**						
Overlooked GFR < 60		10 (47.6%)	4 (19.0%)	5 (23.8%)	9 (42.6%)	4 (19.0%)	8 (38.1%)
Patients with no renal impairment	**21 (77.4%)**						
Wrongly considered to have GFR < 60		2 (2.8%)	12 (16.7%)	6 (8.3%)	4 (5.6%)	9 (12.5%)	5 (6.9%)
Patients with hyperfiltration	**9 (9.7%)**						
Overlooked GFR > 120		2 (22.2%)	9 (100%)	5 (55.6%)	6 (66.7%)	9 (100%)	9 (100%)
Patients with no hyperfiltration	**84 (90.3%)**						
Wrongly considered to have GFR > 120		8 (9.5%)	1 (1.2%)	4 (4.8%)	3 (3.6%)	1 (1.2%)	1 (1.2%)
Total misclassification		22 (23.7%)	26 (28.0%)	20 (21.5%)	22 (23.7%)	23 (24.7%)	23 (24.7%)

Data are given as median [interquartile range] or absolute number (percent; regarding the misclassifications, the percentage of the respective cohort was calculated); bias: difference between mGFR and eGFR; precision: interquartile range of the difference between mGFR and eGFR; accuracy: percentage of eGFR values within 10% (P10) or 30% (P30) of the mGFR; 95% CI: 95% confidence interval

### Detection of impaired kidney function and hyperfiltration

**Fig. 3:**
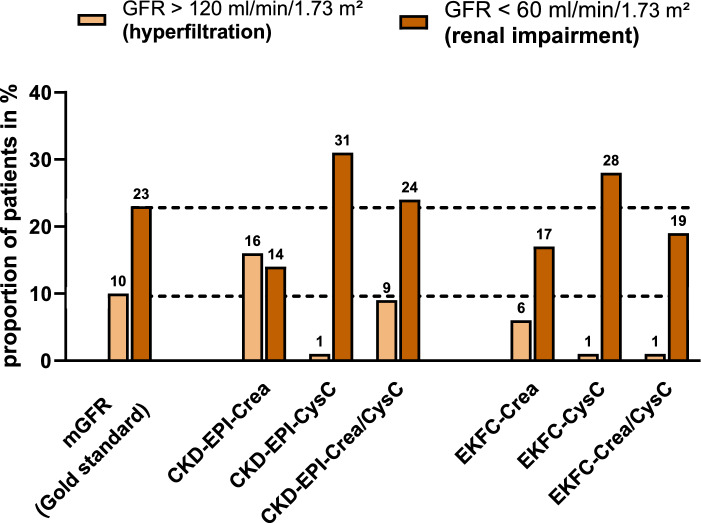
Detection of impaired kidney function and hyperfiltration. Proportion of patients with hyperfiltration, defined as glomerular filtration rate (GFR) > 120 ml/min/1.73 m^2^, and renal impairment (defined as GFR < 60 ml/min/1.73 m^2^) according to measured GFR (mGFR) and estimated GFR (eGFR) formulas. The mGFR-based proportions are depicted as reference values with dotted black lines

Hyperfiltration, defined as GFR ≥ 120 mL/min/1.73 m^2^, was present in 10% of the patients, while 23% had impaired kidney function, defined as GFR < 60 mL/min/1.73 m^2^ (Fig. 3). These proportions were 1–16% and 14–31% for creatinine-based equations, respectively, and 1–6% and 17–28% for cystatin C-based equations (Fig. 3). The combined CKD-EPI-Crea/CysC formula had the best agreement with the proportions detected by mGFR. The proportion of patients with hyperfiltration > 120 ml/min/1.73 m^2^ was significantly higher with the combined CKD-EPI formula (9%) than with the combined EKFC formula (1%, *p* = 0.0346, Fisher's exact test). In sensitivity analyses with a hyperfiltration threshold of > 134 ml/min/1.73m^2^, the creatinine-based CKD-EPI formula showed a significantly higher proportion of patients with hyperfiltration than the EKFC formula. As shown in Table 2, creatinine-based GFR estimation missed the classification of impaired kidney function in 11% (CKD-EPI) and 10% (EKFC) of the cases, respectively. This was only the case in 4% with the cystatin C-based formulas (CKD-EPI/ EKFC). On the other hand, cystatin C-based formulas overlooked hyperfiltration (CKD-EPI/EKFC) in 10% of the cases. Regarding the total misclassification rate, the CKD-EPI_Crea-CysC_ formula showed the best performance with the lowest number of total misclassifications (22%) (Table 2).

### Influence of covariates on the performance of the eGFR formulas

Age, weight, BMI, waist circumference and both the absolute and relative body fat showed a negative correlation with mGFR, while the relative lean tissue mass was positively correlated with mGFR (*p* < 0.05, Supplemental Table 2). Both for the CKD-EPI_CysC_ and the EKFC_CysC_ formula, absolute bias was lower and P30 accuracy was higher in patients with greater lean tissue mass (*p* < 0.05). Patient age was negatively correlated with absolute bias for all formulas (r between -0.27 (95% CI: − 0.44 to − 0.1) and -0.36 (95% CI: − 0.53 to − 0.18), *p* < 0.05). Diabetes type or body fat did not influence the P10 and P30 accuracy of the eGFR formulas.

## Discussion

The present study analyzed the performance of the EKFC and CKD-EPI formula for 93 patients with diabetes mellitus over a broad range of kidney function against a gold standard measurement of GFR using the plasma iohexol clearance. We found that the EKFC_Crea_ formula had a bias closer to zero and a (non-significantly) higher P30 accuracy than the CKD-EPI_Crea_ formula. This is in line with previous results [7, 8]. However, it must be kept in mind that with both formulas, up to a quarter of the patients had an estimated GFR value that varied more than 30% of the measured GFR. Both formulas misclassified patients with impaired renal function in approximately 10% of the cases. For patients with type 2 diabetes, previous studies indicated that the creatinine-based 2009 CKD-EPI formula underestimated GFR in patients with hyperfiltration [15]. In our analysis, the CKD-EPI_Crea_ formula performed better in identifying patients with hyperfiltration than the EKFC_Crea_ formula.

In clinical routine=, cystatin C is gaining interest as an alternative marker for kidney function evaluation. Recently, a joint task force of the National Kidney Foundation and the American Society of Nephrology recommended “national efforts to facilitate increased, routine, and timely use of cystatin C” [16]. In our study, bias and accuracy did not differ significantly for the CKD-EPI_CysC_ and the EKFC_CysC_ formula. This result is consistent with a recent analysis of 6174 Swedish patients, which found no significant difference in the performance of cystatin C-based EKFC and CKD-EPI formula [17]. Our data suggest that both cystatin C-based formulas might perform better than the creatinine-based formulas in the detection of chronic kidney disease and worse in the diagnosis of hyperfiltration. Thus, in clinical routine, the addition of a cystatin C-based GFR estimation might be helpful to identify impaired renal function or chronic kidney disease in patients with diabetes mellitus. Overall, the EKFC_Crea_ formula seemed to perform slightly better than the 2009 CKD-EPI_Crea_ formula in our cohort, while both formulas performed equally for the cystatin C-based GFR estimation.

The creatinine-based formulas differed more than 30% from the cystatin C-based formulas in approximately one in three patients (CKD-EPI formula) and one in six patients (EKFC formula). In these cases, the combined formulas achieved high accuracy and low bias. Therefore, they might be a good option for clinical situations where the true GFR is uncertain. While the combined EKFC formula had a significantly lower bias than the combined CKD-EPI formula, no difference in accuracy was observed. Recently, a study of an overall population of 4050 adults demonstrated better performance of the combined formulas, both for the CKD-EPI and the EKFC formula, than the single marker formulas alone [18]. We confirmed these results in our diabetic subgroup, showing that the combined formulas had the best overall performance. Therefore, they should receive further attention in clinical routine. In line with this, the recently published KDIGO guidelines for the management of chronic kidney disease recommend the use of a combined formula for the estimation of GFR, if cystatin C measurement is available [19].

In an analysis of covariate factors, age and obesity were associated with a decline in measured kidney function. Interestingly, the cystatin C-based formulas had a significantly higher accuracy in patients with greater lean mass. Cystatin C is known to be independent from muscle mass, but potentially influenced by obesity [20]. However, obesity markers such as body fat, BMI, waist circumference or fat tissue index did not have a significant impact on the accuracy of cystatin C-based GFR estimation. Therefore, cystatin C might be used in patients with diabetes mellitus without concern of anthropometric data. In line with our results, a recently published study found a lower accuracy of the cystatin C-based EKFC formula in diabetic patients compared to non-diabetic patients, but traced this difference back to differences in age and GFR levels rather than to the diabetic status [21].

Our results are of high relevance for clinical routine. The creatinine-based eGFR formulas underdiagnosed impaired kidney function in approximately 10% of the cases. Based on the world-wide incidence of 2.62 million cases of diabetes mellitus–related chronic kidney disease in 2019, this would amount to a total of 262,000 patients/year with underdiagnosed chronic kidney disease [22]. Furthermore, this misclassification might lead to a potentially dangerous overdosing of medication that needs to be dosage-adapted based on kidney function (e.g. Metformin, Finerenone). In other cases, important medication might be withheld based on an inaccurately low estimated kidney function. Our data suggest using a combined creatinine- and cystatin C-based formula in European patients with diabetes mellitus to reduce these misclassifications.

Our study has several limitations. First, the sample size was relatively small compared to other studies that investigated the performance of eGFR formulas [23]. For this reason, it is possible that smaller influence factors on GFR measurement might not be detected. In addition, due to the low number of patients with hyperfiltration or impaired kidney function, the results on the detection of these pathologies with different formulas must be interpreted with caution. Especially the number of patients exhibiting hyperfiltration was relatively low. Furthermore, we examined a relatively homogeneous European collective from a single university center. Our results might not be transferable to other populations or subgroups. Regarding the use of the combined formulas, there is a potential risk of propagation of errors due to several variables contained in the formula. While external validation of the plasma iohexol concentrations was carried out by Laboratory Dr. Limbach and Colleagues (Heidelberg, Germany), an external quality control by an Equalis testing program was not performed. Regarding the iohexol clearance measurement, the last sample time point of 210 min after injection might have led to slightly less accurate results compared to longer protocols.

We herein present a real-world, cross-sectional analysis of the performance of the EKFC and the CKD-EPI formulas in patients with diabetes mellitus in a central European cohort against a gold standard measurement of GFR. The best performance was achieved by the combined creatinine- and cystatin C-based formulas. Our findings have direct implications for the daily use of these formulas in clinical routine.

## Supplementary Information

Below is the link to the electronic supplementary material.

Supplementary file1 (PDF 12 KB)

Supplementary file2 (PDF 172 KB)

Supplementary file3 (PDF 197 KB)

Supplementary file4 (PDF 61 KB)

Supplementary file5 (DOCX 26 KB)

## Data Availability

The datasets generated and/or analyzed during the current study are available from the corresponding author on reasonable request.

## References

[1] Sugahara M, Pak WLW, Tanaka T, Tang SCW, Nangaku M (2021) Update on diagnosis, pathophysiology, and management of diabetic kidney disease. Nephrology (Carlton) 26(6):491–500. 10.1111/nep.13860 33550672 10.1111/nep.13860

[2] Costacou T, Orchard TJ (2018) Cumulative kidney complication risk by 50 years of type 1 diabetes: the effects of sex, age, and calendar year at onset. Diabetes Care 41(3):426–433. 10.2337/dc17-1118 28931542 10.2337/dc17-1118 PMC5829956

[3] Levey AS, Stevens LA, Schmid CH, Zhang YL, Castro AF 3rd, Feldman HI et al (2009) A new equation to estimate glomerular filtration rate. Ann Intern Med 150(9):604–612. 10.7326/0003-4819-150-9-200905050-00006 19414839 10.7326/0003-4819-150-9-200905050-00006 PMC2763564

[4] Inker LA, Schmid CH, Tighiouart H, Eckfeldt JH, Feldman HI, Greene T et al (2012) Estimating glomerular filtration rate from serum creatinine and cystatin C. N Engl J Med 367(1):20–29. 10.1056/NEJMoa1114248 22762315 10.1056/NEJMoa1114248 PMC4398023

[5] Inker LA, Eneanya ND, Coresh J, Tighiouart H, Wang D, Sang Y et al (2021) New creatinine- and cystatin c-based equations to estimate GFR without race. N Engl J Med 385(19):1737–1749. 10.1056/NEJMoa2102953 34554658 10.1056/NEJMoa2102953 PMC8822996

[6] Delanaye P, Schaeffner E, Cozzolino M, Langlois M, Plebani M, Ozben T et al (2023) The new, race-free, Chronic Kidney Disease Epidemiology Consortium (CKD-EPI) equation to estimate glomerular filtration rate: is it applicable in Europe? A position statement by the European Federation of Clinical Chemistry and Laboratory Medicine (EFLM). Clin Chem Lab Med 61(1):44–47. 10.1515/cclm-2022-0928 36279207 10.1515/cclm-2022-0928

[7] Pottel H, Delanaye P (2021) Development and validation of a modified full age spectrum creatinine-based equation to estimate glomerular filtration rate. Ann Intern Med 174(7):1038. 10.7326/L21-0248 34280339 10.7326/L21-0248

[8] Pottel H, Bjork J, Rule AD, Ebert N, Eriksen BO, Dubourg L et al (2023) Cystatin C-based equation to estimate GFR without the inclusion of race and sex. N Engl J Med 388(4):333–343. 10.1056/NEJMoa2203769 36720134 10.1056/NEJMoa2203769

[9] Brochner-Mortensen J (1972) A simple method for the determination of glomerular filtration rate. Scand J Clin Lab Invest 30(3):271–274. 10.3109/00365517209084290 4629674 10.3109/00365517209084290

[10] Inker LA, Astor BC, Fox CH, Isakova T, Lash JP, Peralta CA et al (2014) KDOQI US commentary on the 2012 KDIGO clinical practice guideline for the evaluation and management of CKD. Am J Kidney Dis 63(5):713–735. 10.1053/j.ajkd.2014.01.416 24647050 10.1053/j.ajkd.2014.01.416

[11] Tonneijck L, Muskiet MH, Smits MM, van Bommel EJ, Heerspink HJ, van Raalte DH et al (2017) Glomerular hyperfiltration in diabetes: mechanisms, clinical significance, and treatment. J Am Soc Nephrol 28(4):1023–1039. 10.1681/ASN.2016060666 28143897 10.1681/ASN.2016060666 PMC5373460

[12] Ruggenenti P, Porrini EL, Gaspari F, Motterlini N, Cannata A, Carrara F et al (2012) Glomerular hyperfiltration and renal disease progression in type 2 diabetes. Diabetes Care 35(10):2061–2068. 10.2337/dc11-2189 22773704 10.2337/dc11-2189 PMC3447826

[13] Caramori ML, Gross JL, Pecis M, de Azevedo MJ (1999) Glomerular filtration rate, urinary albumin excretion rate, and blood pressure changes in normoalbuminuric normotensive type 1 diabetic patients: an 8-year follow-up study. Diabetes Care 22(9):1512–1516. 10.2337/diacare.22.9.1512 10480518 10.2337/diacare.22.9.1512

[14] Wabel P, Chamney P, Moissl U, Jirka T (2009) Importance of whole-body bioimpedance spectroscopy for the management of fluid balance. Blood Purif 27(1):75–80. 10.1159/000167013 19169022 10.1159/000167013 PMC2813803

[15] MacIsaac RJ, Ekinci EI, Premaratne E, Lu ZX, Seah JM, Li Y et al (2015) The Chronic Kidney Disease-Epidemiology Collaboration (CKD-EPI) equation does not improve the underestimation of Glomerular Filtration Rate (GFR) in people with diabetes and preserved renal function. BMC Nephrol 16:198. 10.1186/s12882-015-0196-0 26630928 10.1186/s12882-015-0196-0 PMC4668645

[16] Delgado C, Baweja M, Crews DC, Eneanya ND, Gadegbeku CA, Inker LA et al (2022) A unifying approach for GFR estimation: recommendations of the NKF-ASN task force on reassessing the inclusion of race in diagnosing kidney disease. Am J Kidney Dis 79(2):268–288. 10.1053/j.ajkd.2021.08.003 34563581 10.1053/j.ajkd.2021.08.003

[17] Fu EL, Levey AS, Coresh J, Grams ME, Faucon AL, Elinder CG et al (2023) Accuracy of GFR-estimating equations based on creatinine, cystatin C or both in routine care. Nephrol Dial Transplant. 10.1093/ndt/gfad219 37813817 10.1093/ndt/gfad219

[18] Inker LA, Tighiouart H, Adingwupu OM, Shlipak MG, Doria A, Estrella MM et al (2023) CKD-EPI and EKFC GFR estimating equations: performance and other considerations for selecting equations for implementation in adults. J Am Soc Nephrol 34(12):1953–1964. 10.1681/ASN.0000000000000227 37796982 10.1681/ASN.0000000000000227 PMC10703072

[19] Kidney Disease: Improving Global Outcomes CKDWG. KDIGO 2024 clinical practice guideline for the evaluation and management of chronic kidney disease. Kidney Int. 2024;105(4):117-314. 10.1016/j.kint.2023.10.018. 10.1016/j.kint.2023.10.018 38490803

[20] Mende CW, Bloomgarden Z (2024) Measurement of renal function: should cystatin C be more widely used for people with diabetes? J Diabetes 16(1):e13534. 10.1111/1753-0407.13534 38282206 10.1111/1753-0407.13534 PMC10822779

[21] Delanaye P, Bjork J, Vidal-Petiot E, Flamant M, Ebert N, Schaeffner E et al (2024) Diabetic status and the performances of creatinine- and cystatin C-based eGFR equations. Nephrol Dial Transplant. 10.1093/ndt/gfae161 39013610 10.1093/ndt/gfae161

[22] Deng Y, Li N, Wu Y, Wang M, Yang S, Zheng Y et al (2021) Global, regional, and national burden of diabetes-related chronic kidney disease from 1990 to 2019. Front Endocrinol (Lausanne) 12:672350. 10.3389/fendo.2021.672350 34276558 10.3389/fendo.2021.672350 PMC8281340

[23] Fu EL, Levey AS, Coresh J, Elinder CG, Rotmans JI, Dekker FW et al (2023) Accuracy of GFR estimating equations in patients with discordances between creatinine and cystatin C-based estimations. J Am Soc Nephrol 34(7):1241–1251. 10.1681/ASN.0000000000000128 36995139 10.1681/ASN.0000000000000128 PMC10356168

[24] Schwertner HA, Weld KJ (2015) High-performance liquid-chromatographic analysis of plasma iohexol concentrations. J Chromatogr Sci 53(9):1475–1480. 10.1093/chromsci/bmv040 25925085 10.1093/chromsci/bmv040

[25] Soman RS, Zahir H, Akhlaghi F (2005) Development and validation of an HPLC-UV method for determination of iohexol in human plasma. J Chromatogr B Analyt Technol Biomed Life Sci 816(1–2):339–343. 10.1016/j.jchromb.2004.11.046 15664368 10.1016/j.jchromb.2004.11.046

