# Supplementary material for: Comparison of GFR estimation in patients with diabetes mellitus using the EKFC and CKD-EPI equations
Source: J Nephrol. 2025 Jan 10;38(2):707–16. doi: 10.1007/s40620-024-02202-4 (PMC11961541; doi:10.1007/s40620-024-02202-4)
Supplement: Supplementary file 1 — Supplementary file1 (PDF 12 KB) [file 40620_2024_2202_MOESM1_ESM.pdf]

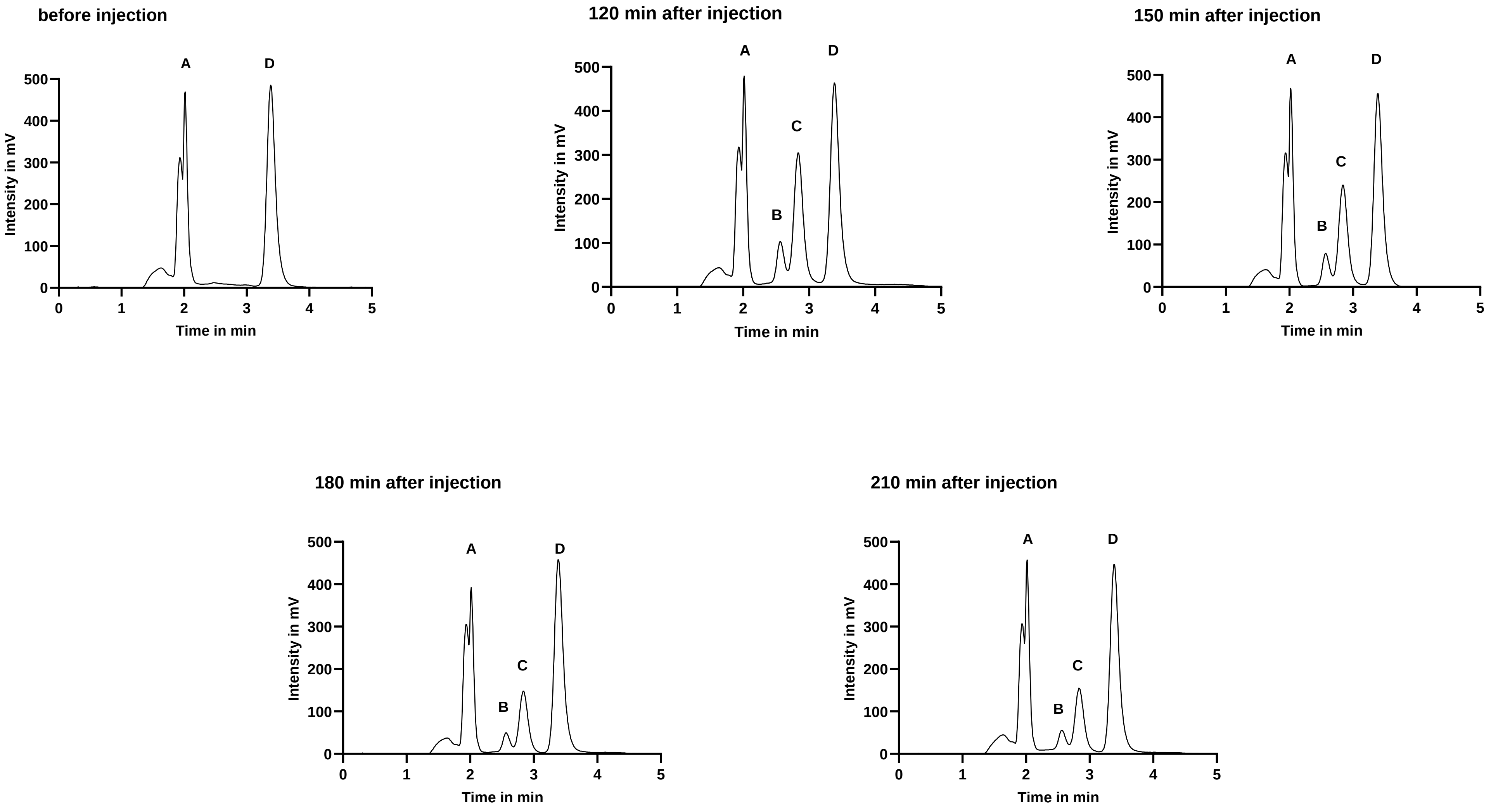

before injection
A
D
Intensity in mV
Time in min
120 min after injection
A
B
C
D
Intensity in mV
Time in min
150 min after injection
A
B
C
D
Intensity in mV
Time in min
180 min after injection
A
B
C
D
Intensity in mV
Time in min
210 min after injection
A
B
C
D
Intensity in mV
Time in min
