# Supplementary figures and images for: Comparison of GFR estimation in patients with diabetes mellitus using the EKFC and CKD-EPI equations
Source: J Nephrol. 2025 Jan 10;38(2):707–16. doi: 10.1007/s40620-024-02202-4 (PMC11961541; doi:10.1007/s40620-024-02202-4)

**A**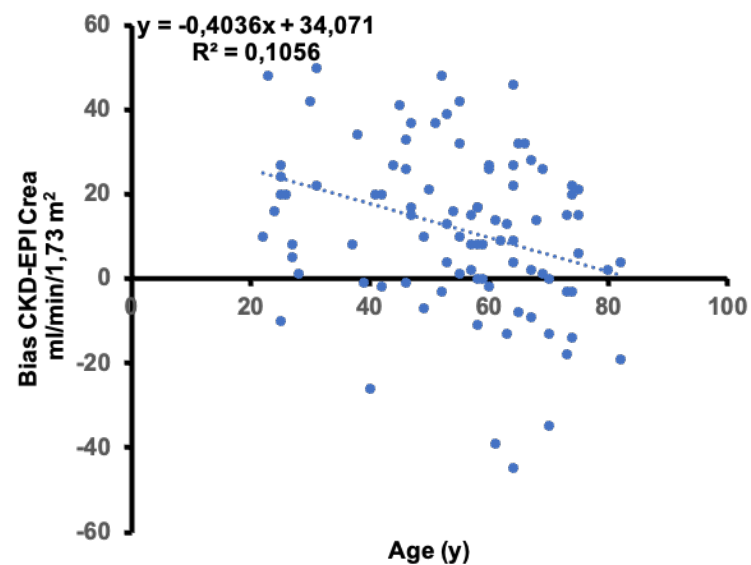**B**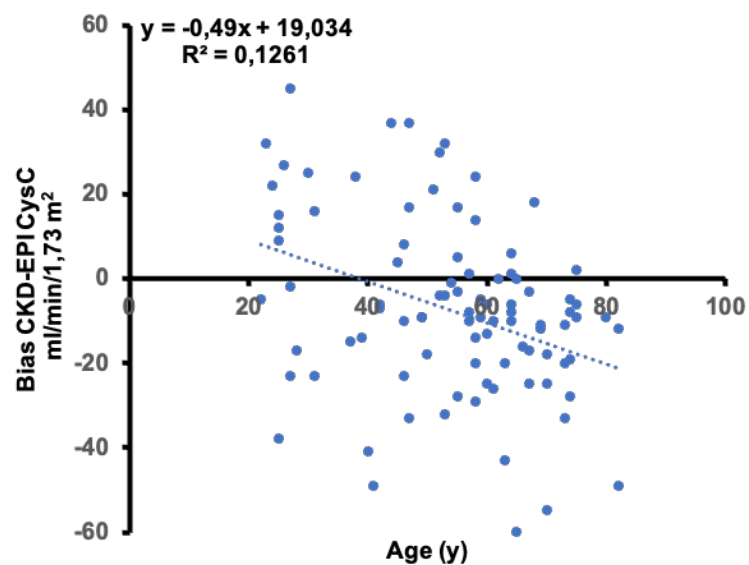**C**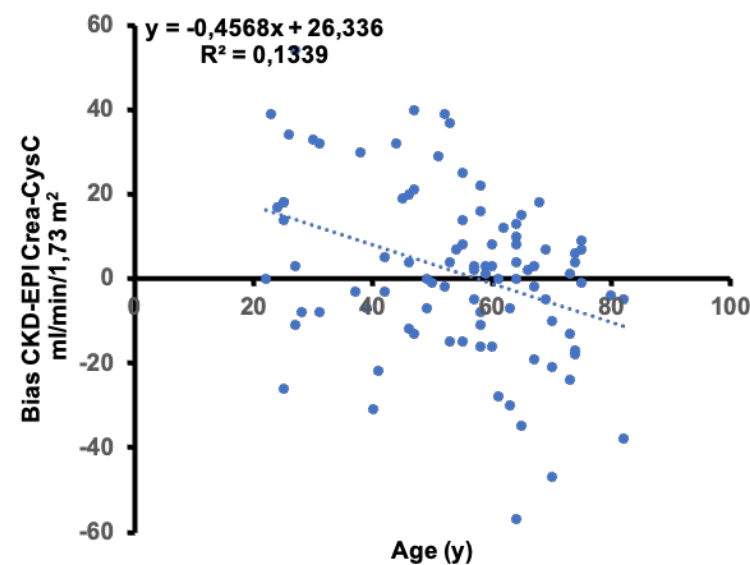**D**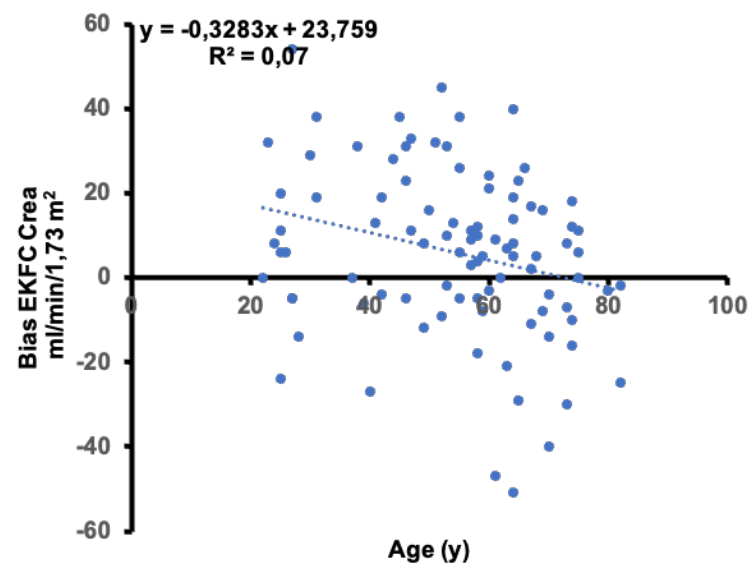**E**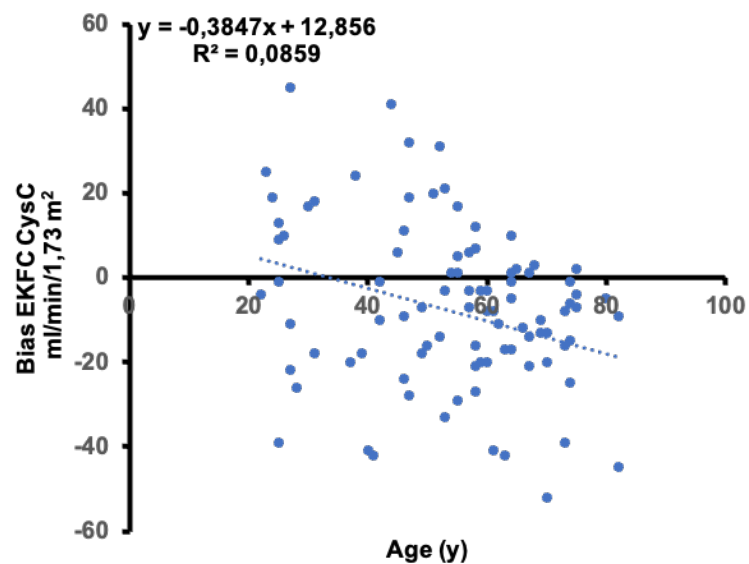**G**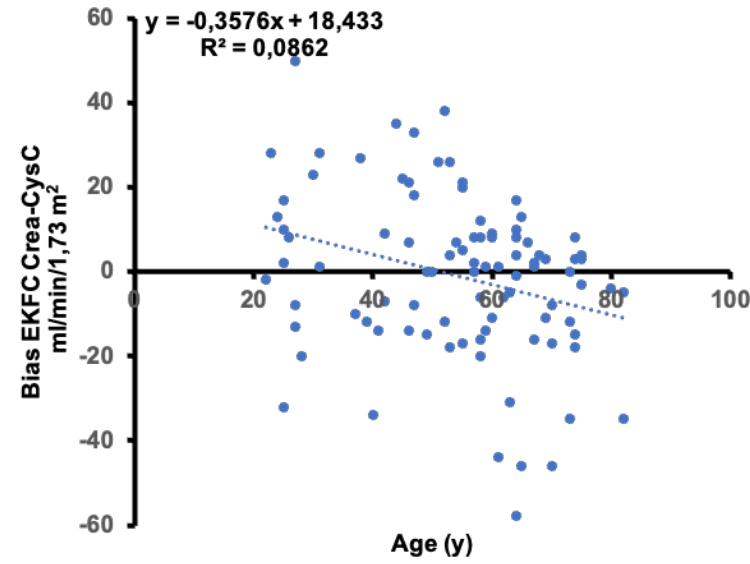

Supplement: Supplementary file 2 — Supplementary file2 (PDF 172 KB) [file 40620_2024_2202_MOESM2_ESM.pdf]

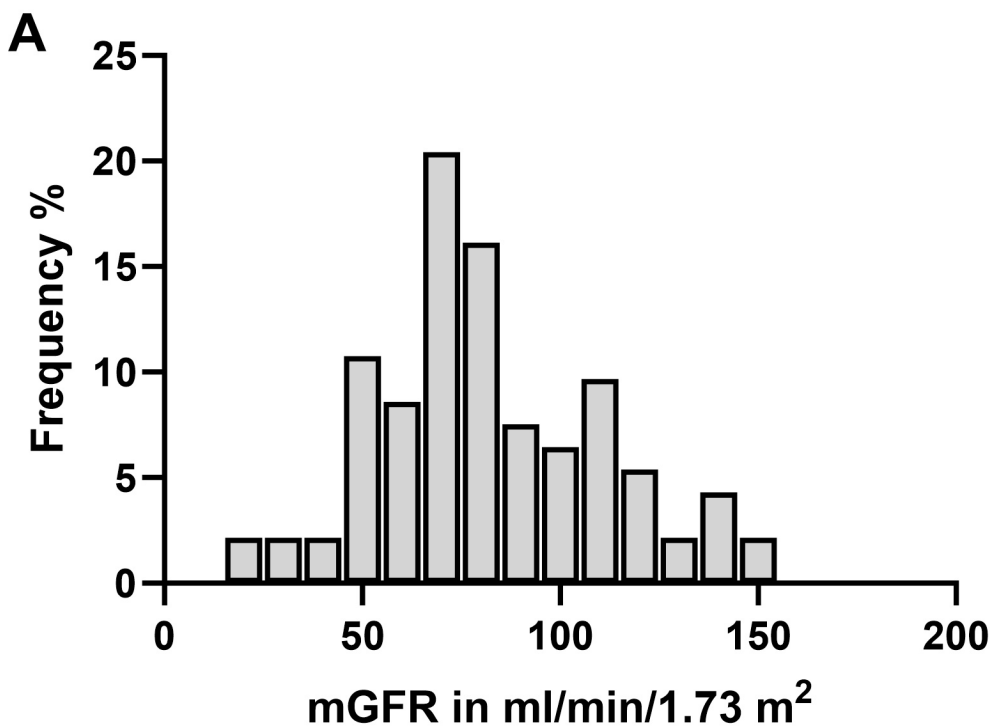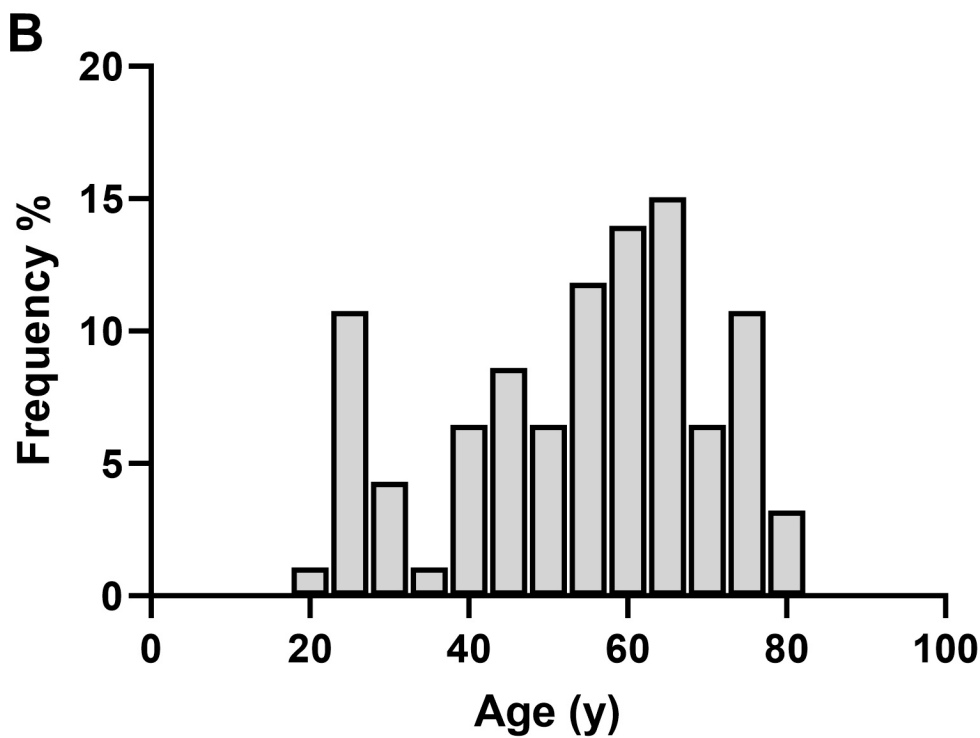

Supplement: Supplementary file 3 — Supplementary file3 (PDF 197 KB) [file 40620_2024_2202_MOESM3_ESM.pdf]

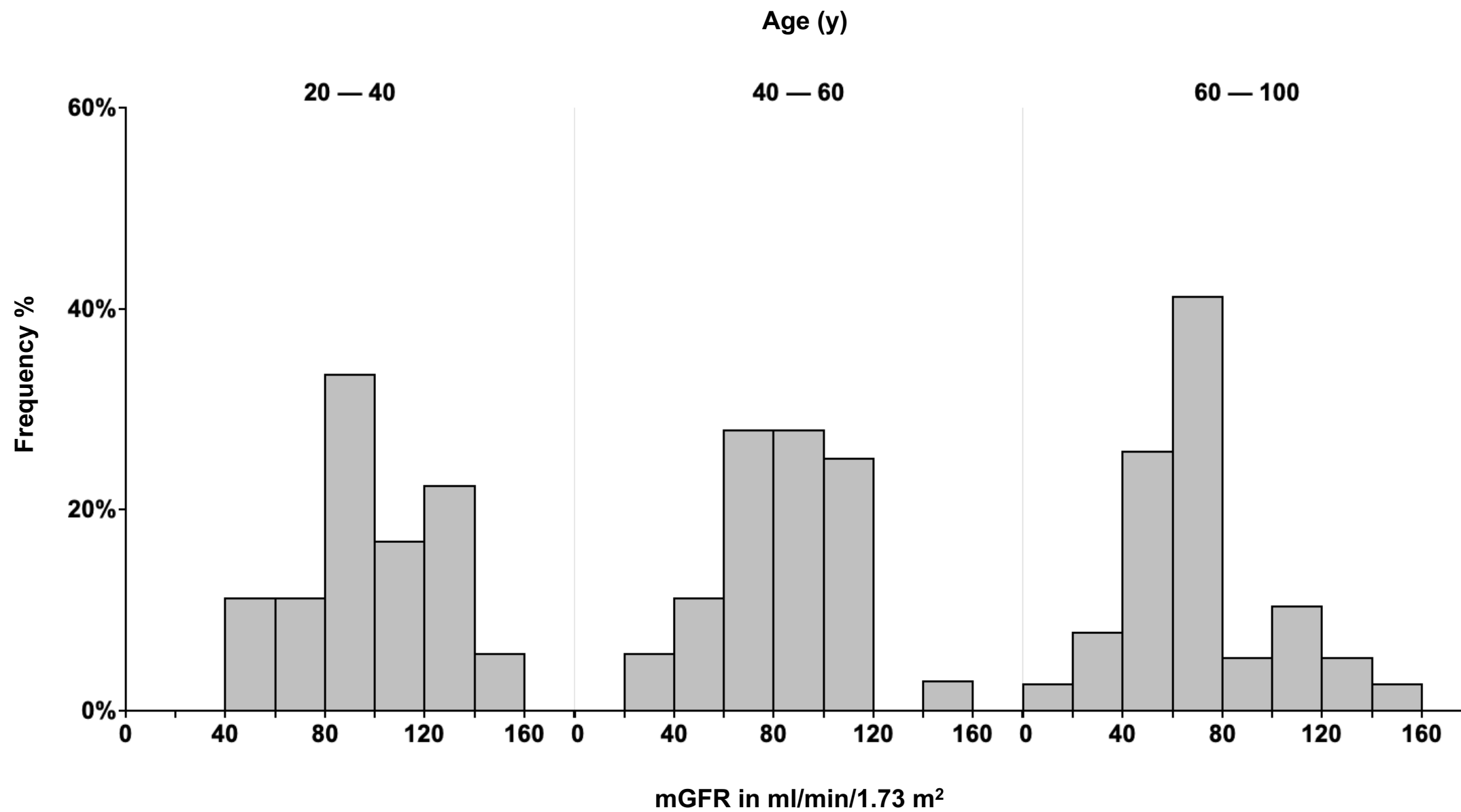

Supplement: Supplementary file 4 — Supplementary file4 (PDF 61 KB) [file 40620_2024_2202_MOESM4_ESM.pdf]
