# Supplementary material for: Comparison of GFR estimation in patients with diabetes mellitus using the EKFC and CKD-EPI equations
Source: J Nephrol. 2025 Jan 10;38(2):707–16. doi: 10.1007/s40620-024-02202-4 (PMC11961541; doi:10.1007/s40620-024-02202-4)
Supplement: Supplementary file 5 — Supplementary file5 (DOCX 26 KB) [file 40620_2024_2202_MOESM5_ESM.docx]

**Supplemental Figure 1: Representative chromatograms from HPLC for the determination of the plasma iohexol concentration**

Representative chromatograms of blood plasma samples before and 120, 150, 180 and 210 minutes after injection of iohexol. Retention time is shown on the x-axis, signal intensity on the y-axis. Peaks B and C represent the characteristic double peak of iohexol. Peak A (plasma background) and peak D (internal standard) are baseline peaks that can also be found in blood plasma samples before injection of iohexol.

**Supplemental Figure 2: Scatter plot of bias and age**

A-F) Scatter plot depicting the relationship between the bias of eGFR formulas and age. The trendline is given in blue. Coefficient of determination (R^2^) and equation of the trend line are given.

**Supplemental Figure 3: Histograms of age and mGFR**

Illustration of the distribution of mGFR (A) and age (B) in the study population. The x-axis represents mGFR values in mL/min/1.73 m² (A) or age in years (B) and the y-axis represents the frequency of participants.

**Supplemental Figure 4: Combined Histogram of age and mGFR**

Histogram showing the distribution of age and mGFR in the study population. The x-axis represents mGFR values (mL/min/1.73 m²) and the y-axis represents the frequency of participants within each group. Overlaid on the histogram are the age groups, illustrating the relationship between age and renal function in the cohort.

**Supplemental Table 1: BCM results**

| Overhydration (litre) | 0.4 [-0.6 – 1.5] |
| --- | --- |
| Overhydration (%) | 2.3 [-3.3 – 8.1] |
| Total Body Water (TBW) (litre) | 38.9 [33.1 – 48.2] |
| Extracellular Water (ECW) (litre) | 18.5 [16.2 – 20.7] |
| Intracellular Water (ICW) (litre) | 20.5 [16.7 – 23.4] |
| ECW / ICW | 0.9 [0.8 – 1] |
| Lean tissue index (LTI) (kg/m^2^) | 12.3 [10.7 – 14.2] |
| Fat tissue index (FTI) (kg/m^2^) | 14.9 [11.7 – 21] |
| Lean tissue mass (LTM) (kg) | 37.9 [30.4 – 44.5] |
| Lean tissue mass (LTM) (%) | 44.6 [33.1 – 51.8] |
| Fat mass (kg) | 34.1 [26.7 – 44.2] |
| Fat mass (%) | 39.6 [33.8 – 47.6] |
| Adipose tissue mass (ATM) (kg) | 46.2 [36.4 – 60] |
| Body cell mass (kg) | 20.1 [16.2 – 24.7] |

*Data are given as median [interquartile range]*

**Supplemental Table 2: Univariate, pairwise correlation of body composition parameters with GFR**

|  | **mGFR** | **CKD- EPI Crea 2009** | **CKD- EPI CysC 2012** | **CKD- EPI Crea-Cys 2012** | **EKFC Crea 2021** | **EKFC CysC 2023** | **EKFC Crea-Cys 2023** |
| --- | --- | --- | --- | --- | --- | --- | --- |
| Age (y) | -0.38 (p=0.0002) | -0.62 (p<0.0001) | -0.64 (p<0.0001) | -0.63 (p<0.0001) | -0.64 (p<0.0001) | -0.68 (p<0.0001) | -0.68 (p<0.0001 |
| Height (m) | -0.03 (p=0.74) | 0.16 (p=0.12) | 0.05 (p=0.67) | 0.08 (p=0.47) | 0.18 (p=0.09) | 0.08 (p=0.45) | 0.13 (p=0.2) |
| Weight (kg) | -0.28 (p=0.007) | -0.08 (p=0.43) | -0.26 (p=0.01) | -0.21 (p=0.048) | -0.06 (p=0.6) | -0.23 (p=0.03) | -0.15 (p=0.16) |
| BMI (kg/m²) | -0.31 (p=0.003) | -0.17 (p=0.1) | -0.32 (p=0.002) | -0.27 (p=0.008) | -0.15 (p=0.15) | -0.3 (p=0.003) | -0.23 (p=0.02) |
| HbA1c (%) | 0.07 (p=0.52) | 0.06 (p=0.56) | 0.003 (p=0.98) | 0.03 (p=0.78) | 0.07 (p=0.49) | -0.008 (p=0.99) | 0.03 (p=0.73) |
| Overhydration (litre) | 0.11 (p=0.32) | 0.02 (p=0.86) | -0.03 (p= 0.75) | -0.01 (p= 0.9) | 0.003 (p= 0.97) | -0.06 (p= 0.54) | -0.03 (p= 0.76) |
| Waist circumference (cm) | -0.37 (p=0.003) | -0.27 (p=0.01) | -0.46 (p<0.0001) | -0.39 (p=0.0001) | -0.25 (p=0.018) | -0.44 (p<0.0001) | -0.35 (p=0.0006) |
| Total Body Water (TBW) (litre) | -0.16 (p=0.14) | 0.1 (p=0.37) | -0.09 (p= 0.41) | -0.03 (p= 0.76) | 0.13 (p= 0.23) | -0.05 (p= 0.63) | 0.04 (p= 0.7) |
| Extracellular Water (ECW) (litre) | -0.11 (p=0.28) | -0.1 (p=0.35) | -0.24 (p= 0.02) | -0.2 (p= 0.06) | -0.08 (p= 0.43) | -0.23 (p= 0.03) | -0.16 (p= 0.13) |
| Intracellular Water (ICW) (litre) | -0.08 (p=0.42) | 0.05 (p=0.65) | -0.09 (p= 0.41) | -0.05 (p= 0.63) | 0.07 (p= 0.54) | -0.06 (p= 0.56) | 0.003 (p= 0.98) |
| ECW / ICW | -0.2 (p=0.057) | -0.35 (p=0.0006) | -0.43 (p<0.0001) | -0.39 (p=0.0001) | -0.37 (p=0.0003) | -0.46 (p<0.0001) | -0.43 (p<0.0001) |
| Lean tissue index (LTI) (kg/m^2^) | -0.09 (p=0.38) | 0.04 (p=0.7) | 0.1 (p=0.36) | 0.07 (p=0.52) | 0.03 (p=0.75) | 0.1 (p=0.34) | 0.07 (p=0.5) |
| Fat tissue index (FTI) (kg/m^2^) | -0.27 (p=0.01) | -0.22 (p=0.04) | -0.32 (p=0.0021) | -0.28 (p=0.007) | -0.2 (p=0.056) | -0.31 (p=0.003) | -0.27 (p=0.01) |
| Lean tissue mass (LTM) (kg) | 0.01 (p=0.92) | 0.23 (p=0.03) | 0.22 (p=0.03) | 0.21 (p=0.04) | 0.24 (p=0.02) | 0.24 (p=0.02) | 0.25 (p=0.02) |
| Lean tissue mass (LTM) (%) | 0.21 (p=0.04) | 0.26 (p=0.01) | 0.39 (p=0.0001) | 0.34 (p=0.0008) | 0.26 (p=0.01) | 0.39 (p=0.0001) | 0.34 (p=0.001) |
| Fat mass (kg) | -0.29 (p=0.046) | -0.2 (p=0.056) | -0.34 (p=0.001) | -0.29 (p=0.005) | -0.18 (p=0.08) | -0.32 (p=0.002) | -0.26 (p=0.01) |
| Fat mass (%) | -0.22 (p=0.036) | -0.24 (p=0.02) | -0.3 (p=0.004) | -0.27 (p=0.008) | -0.24 (p=0.02) | -0.3 (p=0.004) | -0.28 (p=0.007) |
| Adipose tissue mass (kg) | -0.29 (p=0.006) | -0.19 (p=0.07) | -0.32 (p=0.001) | -0.28 (p=0.007) | -0.17 (p=0.1) | -0.31 (p=0.003) | -0.25 (p=0.018) |

*Data are given as correlation coefficients (p-value), grey highlighted sections represent values below the level of significance (p ≤ 0.05)*

**Supplementary Information: Description of the eGFR formulas**

The following formulas were used (SCr: serum creatinine, SCysC: serum cystatin C):

2009 CKD-EPI_crea_ formula:

Female

If serum creatinine level ≤ 0.70 mg/dl: GFR = 144 × (SCr/0.70)^−0.329^ × 0.9929^Age^ × 1.159

If serum creatinine level > 0.70 mg/dl: GFR = 144 × (SCr/0.70)^−1.209^ × 0.9929^Age^ × 1.159

Male

If serum creatinine level ≤ 0.90 mg/dl: GFR = 141 × (SCr/0.90)^−0.411^ × 0.9929^Age^ × 1.159

If serum creatinine level > 0.90 mg/dl: GFR = 141 × (SCr/0.90)^−1.209^ × 0.9929^Age^ × 1.159

2012 CKD-EPI_CysC_ formula:

Female

If serum cystatin C level ≤ 0.80 mg/l: GFR = 133 × (SCysC/0.80)^−0.499^ × 0.9962^Age^ × 0.932

If serum cystatin C level > 0.80 mg/l: GFR = 133 × (SCysC/0.80)^−1.328^ × 0.9962^Age^ × 0.932

Male

If serum cystatin C level ≤ 0.80 mg/l: GFR = 133 × (SCysC/0.80)^−0.499^ × 0.9962^Age^

If serum cystatin C level > 0.80 mg/l: GFR = 133 × (SCysC/0.80)^−1.328^ × 0.9962^Age^

2012 CKD-EPI_Crea-CysC_ formula:

Female

If serum creatinine level ≤ 0.70 mg/dl and serum cystatin C level ≤ 0.80 mg/l:

GFR = 130 × (SCr/0.70)^−0.248^ × (ScysC/0.80)^−0.375^ × 0.9952^Age^

If serum creatinine level ≤ 0.70 mg/dl and serum cystatin C level > 0.80 mg/l:

GFR = 130 × (SCr/0.70)^−0.248^ × (ScysC/0.80)^−0.711^ × 0.9952^Age^

If serum creatinine level > 0.70 mg/dl and serum cystatin C level ≤ 0.80 mg/l:

GFR = 130 × (SCr/0.70)^−0.601^ × (ScysC/0.80)^−0.375^ × 0.9952^Age^

If serum creatinine level > 0.70 mg/dl and serum cystatin C level > 0.80 mg/l:

GFR = 130 × (SCr/0.70)^−0.601^ × (ScysC/0.80)^−0.711^ × 0.9952^Age^

Male

If serum creatinine level ≤ 0.90 mg/dl and serum cystatin C level ≤ 0.80 mg/l:

GFR = 135 × (SCr/0.90)^−0.207^ × (ScysC/0.80)^−0.375^ × 0.9952^Age^

If serum creatinine level ≤ 0.90 mg/dl and serum cystatin C level > 0.80 mg/l:

GFR = 135 × (SCr/0.90)^−0.207^ × (ScysC/0.80)^−0.711^ × 0.9952^Age^

If serum creatinine level > 0.90 mg/dl and serum cystatin C level ≤ 0.80 mg/l:

GFR = 135 × (SCr/0.90)^−0.601^ × (ScysC/0.80)^−0.375^ × 0.9952^Age^

If serum creatinine level > 0.90 mg/dl and serum cystatin C level > 0.80 mg/l:

GFR = 135 × (SCr/0.90)^−0.601^ × (ScysC/0.80)^−0.711^ × 0.9952^Age^

2021 EKFC_Crea_ formula:

Female

Age 18-40 years, serum creatinine level/Q value <1.0:

GFR = 107.3 × (SCr/Q)^−0.322^

Age 18-40 years, serum creatinine level/Q value ≥1.0:

GFR = 107.3 × (SCr/Q)^−1.132^

Age >40 years, serum creatinine level/Q value <1.0:

GFR = 107.3 × (SCr/Q)^−0.322^ × 0.990^(Age–40)^

Age 18-40 years, serum creatinine level/Q value ≥1.0:

GFR = 107.3 × (SCr/Q)^−1.132^ × 0.990^(Age–40)^

Male

Age 18-40 years, serum creatinine level/Q value <1.0:

GFR = 107.3 × (SCr/Q)^−0.322^

Age 18-40 years, serum creatinine level/Q value ≥1.0:

GFR = 107.3 × (SCr/Q)^−1.132^

Age >40 years, serum creatinine level/Q value <1.0:

GFR = 107.3 × (SCr/Q)^−0.322^ × 0.990^(Age–40)^

Age 18-40 years, serum creatinine level/Q value ≥1.0:

GFR = 107.3 × (SCr/Q)^−1.132^ × 0.990^(Age–40)^

Q value White Europeans: male = 0.90 female = 0.70

2021 EKFC_CysC_ formula:

Male and Female

Age 18-40 years, serum creatinine level/Q value <1.0:

GFR = 107.3 × (SCysC/Q)^−0.322^

Age 18-40 years, serum creatinine level/Q value ≥1.0:

GFR = 107.3 × (SCysC/Q)^−1.132^

Age >40 years, serum creatinine level/Q value <1.0:

GFR = 107.3 × (SCysC/Q)^−0.322^ × 0.990^(Age-40)^

Age 18-40 years, serum creatinine level/Q value ≥1.0:

GFR = 107.3 × (SCysC/Q)^−1.132^ × 0.990^(Age-40)^

Q value: Q = 0.83 until 50 years, Q = 0.83 + 0.005 x (Age−50) after 50 years

2023 EKFC_Crea-CysC_ formula:

Arithmetic mean of EKFC_Crea_ and EKFC_CysC_

**Supplementary Information: Iohexol measurement**

The iohexol plasma concentration was determined using High Pressure Liquid Chromatography (HPLC) with modifications of previously described protocols [24, 25]. Initially, 100 µl of EDTA plasma was mixed with 100 µL of pre-cooled precipitant (acetonitrile - ethanol - water in a volumetric ratio of 60.0% - 38.4% - 1.6%) and refrigerated at 4 °C for 2 hours. This was followed by centrifugation at 14,100 g for 10 minutes at 4 °C to pelletize the proteins. The supernatant was then transferred to fresh tubes, and centrifugation was repeated under the same conditions. The clear supernatant was finally transferred to new tubes for subsequent analysis and stored at 4 °C for short-term use on the day of the experiment, and at -20 °C for longer storage. Before HPLC measurements, it was centrifuged again for 1 minute at 14,100 g to pellet any residual particulates. For HPLC analysis, 5 μL of this supernatant was added to 391 μL of mobile phase (HPLC-grade water with 7 % acetonitrile and 0.055% trifluoroacetic acid (TFA)) and 4 µl of internal standard iohexol related compound B (100 µg/mL, resulting in a final concentration of 1 µg/mL). A series of iohexol standards were prepared with concentrations of 1, 1.5, 2, and 2.5 µg/mL, diluted in healthy control plasma. The sample injection volume at the autosampler was set to 50 µL, and the flow rate adjusted to 0.9 mL/min. The mobile phase was maintained under isocratic conditions without a gradient. For the chromatographic separation, a 250 mm length C-18 reversed-phase column with 4.6 mm inner diameter was used (Spherisorb ODS-2, 5 µm, Grom, Rottenburg-Hailfingen, Germany). The chromatographic eluates were monitored and quantified by an Ultraviolet-Visible (UV-Vis) detector. Iohexol was identified by its characteristic double peak at the extinction wavelength of 254 nm (refer to **Suppl. Figure 1**). Peak quantification was performed based on the area under the curve (AUC), using the ChromStar 7 software (SCPA, Weyhe-Leeste, Germany). The obtained plasma iohexol concentrations were externally validated by determination of the plasma iodine concentration using mass spectrometry (Labor Dr. Limbach and Colleagues, Heidelberg, Germany).
